# Prone Vs. Supine Position Ventilation in Intubated COVID-19 Patients: A Systematic Review and Meta-Analysis

**DOI:** 10.7759/cureus.39636

**Published:** 2023-05-29

**Authors:** Mohamed Fayed, Wissam Maroun, Ahmed Elnahla, Nicholas Yeldo, Jessica R Was, Donald H Penning

**Affiliations:** 1 Anesthesiology, Pain Management and Perioperative Medicine, Henry Ford Health System, Detroit, USA

**Keywords:** severe sepsis, severe respiratory failure, covid-19 mortality, icu mortality rate, systematic review and meta analysis, invasive mechanical ventilation, acute respiratory distress syndrome [ards], supine position, prone positioning, covid 19

## Abstract

Whether prone positioning of patients undergoing mechanical ventilation for COVID-19 pneumonia has benefits over supine positioning is not clear. We conducted a systematic review with meta-analysis to determine whether prone versus supine positioning during ventilation resulted in different outcomes for patients with COVID-19 pneumonia. We searched Ovid Medline, Embase, and Web of Science for prospective and retrospective studies up through April 2023. We included studies that compared outcomes of patients with COVID-19 after ventilation in prone and supine positions. The primary outcomes were three mortality measures: hospital, overall, and intensive care unit (ICU). Secondary outcomes were mechanical ventilation days, intensive care unit (ICU) length of stay, and hospital length of stay. We conducted risk of bias analysis and used meta-analysis software to analyze results. Mean difference (MD) was used for continuous data, and odds ratio (OR) was used for dichotomous data, both with 95% CIs. Significant heterogeneity (I^2^) was considered if I^2^ was >50%. A statistically significant result was considered if the p-value was <0.05. Of 1787 articles identified, 93 were retrieved, and seven retrospective cohort studies encompassing 5216 patients with COVID-19 were analyzed. ICU mortality was significantly higher in the prone group (OR 2.22, 95% CI 1.43-3.43; p=0.0004). No statistically significant difference was observed between prone and supine groups for hospital mortality (OR, 0.95; 95% CI, 0.66-1.37; p=0.78) or overall mortality (OR, 1.08; 95% CI, 0.72-1.64; p=0.71). Studies that analyzed primary outcomes had significant heterogeneity. Hospital length of stay was significantly higher in the prone than in the supine group (MD, 6.06; 95 % CI, 3.15-8.97; p<0.0001). ICU length of stay and days of mechanical ventilation did not differ between the two groups. In conclusion, mechanical ventilation with prone positioning for all patients with COVID-19 pneumonia may not provide a mortality benefit over supine positioning.

## Introduction and background

Acute respiratory distress syndrome (ARDS) is a condition characterized by widespread patchy and coalescent airspace opacities in the lungs as seen by computed tomography (CT); these are usually more apparent in the dependent lung zones. This can lead to a ventilation and perfusion mismatch with resultant severe hypoxemia and respiratory failure [1,2]. Prone ventilation is an effective treatment strategy for patients with ARDS [3,4]. By placing the patient in a prone position, the ventral-dorsal trans-pulmonary pressure difference is ameliorated, which helps reduce dorsal lung compression and improve lung perfusion [5-7]. This leads to improving the ventilation and perfusion mismatch and hence oxygenation [2,5,8,9]. However, the prone positioning in severe ARDS (PROSEVA) trial had several limitations, including the exclusion of a significant proportion of patients, and it was conducted at a center experienced in prone ventilation [4].

Coronavirus disease 2019 (COVID-19) pneumonia differs from classic ARDS in several ways. Autopsy reports of patients who have died from COVID-19 pneumonia have described hyaline membrane changes and micro-vessel thrombosis, which are not commonly seen in classic ARDS [10-13]. The anatomical location of COVID-19 pneumonia is also different, with bilateral, peripheral, and multilobar ground-glass opacities being the most common CT manifestations rather than involving the dependent lung zones [14]. Hence, the location of ground-grass opacities might not be as responsive to prone positioning in improving the ventilation and perfusion mismatch. Regarding the use of prone positioning during ventilation for patients with COVID-19 pneumonia, one study found no statistically significant variation in the respiratory system and lung mechanics in a prone position. This finding suggests that lung aeration is generally preserved in patients with COVID-19 pneumonia and may even tend to worsen because of prone ventilation [15]. This also suggests that lung mechanics during COVID-19 pneumonia may appear normal despite severe lung damage and that the trans-pulmonary pressures may remain below harmful thresholds [15].

Given the pathophysiological differences between patients with COVID-19 pneumonia and those with classic ARDS, it is reasonable to hypothesize that the efficacy of prone ventilation may be different in patients with COVID-19. Therefore, we systematically synthesized current evidence on the mortality outcomes resulting from prone-position mechanical ventilation compared to supine-position mechanical ventilation for patients with COVID-19 pneumonia.

This article was previously presented as a meeting abstract at the 2023 Society of Critical Care Congress on January 23, 2023 [16].

## Review

Methods 

We conducted a systematic review and meta-analysis adhering to the preferred reporting items for systematic reviews and meta-analyses (PRISMA) 2020 [17,18]. Our study aimed to explore the association of prone versus supine ventilation with outcomes in patients with COVID-19 pneumonia. Primary outcomes included three mortality measures: ICU mortality, hospital mortality, and overall mortality. Secondary outcomes included mechanical ventilation days, intensive care unit (ICU) length of stay, and hospital length of stay. We considered the last follow-up point of mortality as overall mortality.

Literature Search Strategy

**Figure 1:**
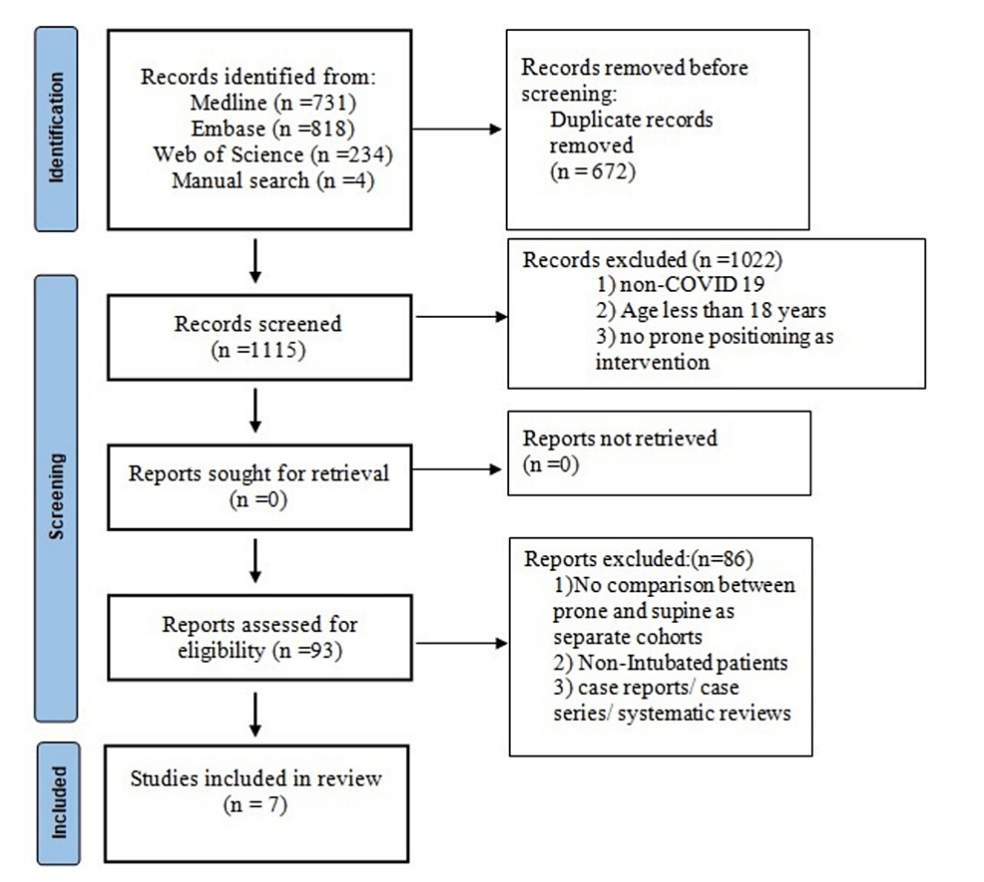
PRISMA Flow Diagram PRISMA: preferred reporting items for systematic reviews and meta-analyses, n: number

We thoroughly searched three databases for articles: Ovid Medline, Embase, and Web of Science. We sought articles that compared outcomes in patients with COVID-19 who underwent mechanical ventilation in the prone versus supine position. A systematic search strategy using medical subject headings and keywords was developed to extract all the relevant articles for analysis. The keywords included “COVID-19,” “SARS-COV-2,” “Prone Position,” “Proning,” “Patient positioning,” “Intubation,” and “Mechanical ventilation.” Additional inclusion criteria included papers published up through April 2023 and papers published in English. We excluded articles that involved patients less than 18 years old. The detailed search strategy is shown in Figure 1.

Data Extraction

Articles were independently screened for relevance by two authors per title, abstract, and full text. In cases of discrepancy between the reviewers, a third reviewer was consulted, and conflicts were resolved through discussion by a mutual understanding of the inclusion and exclusion criteria. The information extracted from the final chosen articles included the year of publication, patient demographics, comorbidities, COVID-19-associated ARDS severity, sequential organ failure assessment (SOFA) score, acute physiological and chronic health evaluation (APACHE II) score, ICU mortality, hospital mortality, overall mortality, ICU length of stay, hospital length of stay, and mechanical ventilation days. Patients in the studies were divided into two groups based on ventilation positioning: a supine group and a prone group. Data were extracted and analyzed in Microsoft Excel (Microsoft Corporation, Redmond, WA).

Risk of Bias in Individual Studies

The risk of bias for all included observational studies was independently assessed by two authors using the Newcastle-Ottawa Scale [19]. Studies with three or four stars in the selection domain, one or two stars in the comparability domain, and two or three stars in the outcome/exposure domain were considered good quality studies with a low risk of bias. The certainty of the evidence was conducted independently by two authors. Conflicts were discussed with a third author (Table 1).

**Table 1:** Certainty of Evidence Assessment CI: confidence interval, OR: odds ratio, MD: mean difference, ICU: intensive care unit,^ a^ Based on I^2^ is more than 50, ^b^ Different follow-up periods and different prone criteria, ^c^ based on wide confidence intervals

Certainty Assessment							Number of patients		Effect	Certainty
Number of studies	Studies Design	Risk Of biases	Inconsistency	Indirectness	Imprecision	Other considerations	Prone	Supine	Relative (95% CI)	
ICU Mortality										
2	Retrospective Cohorts	Not serious	Very serious ^a^	Serious ^b^	Serious ^c^	None	921	687	OR 2.22(1.43-3.43)	Very Low
Hospital Mortality										
6	Retrospective Cohorts	Not serious	Very serious ^a^	Serious ^b^	Serious ^c^	None	1993	2672	OR 0.95(0.66-1.37)	Very Low
Overall Mortality										
7	Retrospective Cohorts	Not serious	Very serious ^a^	Serious ^b^	Serious ^c^	None	2266	2950	OR 1.08(0.72-1.64)	Very Low
Length of Hospital Stay										
4	Retrospective Cohorts	Not serious	Very serious ^a^	Serious ^b^	Serious ^c^	None	1268	1019	MD 6.06(3.15-8.97)	Very Low
ICU length of stay										
3	Retrospective Cohorts	Not serious	Very serious	Serious	Serious	None	1206	820	MD 2.28(-1.17-5.74)	Very Low
Days of Mechanical Ventilation										
2	Retrospective Cohorts	Not serious	Very serious	Serious	Serious	None	1086	705	MD 3.28(-4.02-10.58)	Very Low

Statistical Analysis

We used the ReviewManager software (Copenhagen: The Nordic Cochrane Centre, The Cochrane Collaboration) to analyze the results. The mean difference (MD) was used to analyze continuous data while the odds ratio (OR) was used to analyze dichotomous data. Both estimates were reported with a 95% confidence interval (CI). We considered significant heterogeneity if I^2^ was >50. We considered p value <0.05 as a statistically significant result. A random model was used for those data [17]. Publication bias and meta-regression were not performed because the total number of studies for analyzing all outcomes was less than 10.

Results

Study Selection

The study selection process is summarized in the PRISMA flow diagram (Figure 1). The search strategy provided 1787 articles for the title and abstract screening, of which 93 were retrieved for full-text screening. After inclusion and exclusion criteria were applied, seven retrospective cohort studies that encompassed 5216 patients with COVID-19 were included in the analysis [20-26].

Study Characteristics

There were two studies that were conducted in the United States (US), and the remaining five were conducted in the United Kingdom, China, Jordan, Netherlands, and Italy (Table 2). Patient inclusion criteria were well-defined in the selected studies; however, prone position duration and frequency and ventilation strategy were not consistently defined. All studies were retrospective cohort studies. In five studies, the supine group was older than the prone group, while both groups had similar ages in one study, and age was not reported in one study [24]. In four studies, the prone group included proportionately more men than the supine group, while two studies had a higher proportion of men in the supine group, and sex was not reported in one study. APACHE II scores were reported in five studies, and SOFA scores were reported in four studies. The prone group had a higher median APACHE II score than the supine group in four studies [21,23,25,26] and a lower median score in one study [20]. SOFA score was similar in two studies [21,22] and was higher in the prone group in the other studies [25,26]. In five studies that reported the prevalence of COVID-19-associated ARDS, the prone group had a higher severe ARDS rate than the supine group (Table 2). 

**Table 2:** Baseline Characteristics of Selected Studies *: Mean and standard deviation, P: prone, S: supine, ARDS: adult respiratory distress syndrome, CRD: chronic respiratory disease, DM: diabetes mellitus, IC: immunocompromised, CAD: coronary artery disease, CKD: chronic kidney disease, NR: not reported Source: references [20-26]

Study ID	Study design	Country	Compared groups	Sample size	Age (median (IQR))	Male (%)	BMI (median (IQR))	APACHE II (median (IQR))	Severe ARDS (%)	CRD (%)	DM or IC (%)	Smoker (%)	CAD (%)	CKD (%)
Chen et al, 2021 [20]	Retrospective cohort	China	P/S	23/17	69(56–87)/ 72(54–89) *	64.7/78.3	NR	15(8–38)/16(8–25) *	71/61	5.9/8.7	23.5/34.8	5.9/8.7	11.8/8.7	5.9/8.7
Langer et al., 2021 [21]	Retrospective cohort	Italy	P/S	648/409	63(55-69)/ 63(55-69)	79/78	28(25-31)/27(25-31)	10(8-13)/9(7-13)	35/16.9	NR	NR	NR	NR	NR
Mathews et al., 2021 [22]	Retrospective cohort	USA	P/S	702/1636	60(51-69)/ 63(53-72)	67.5/64.4	31.5(27.4-37.2)/ 30.6(26.7-35.9)	NR	57/47.2	19.2/22.4	NR	NR	10.4/13.9	NR
Shelhamer et al., 2021 [23]	Retrospective cohort	USA	P/S	62/199	60(54.3-66.5)/ 66(55-74.5)	67.7/60.3	30.9 (28.3-35.9)/31(26.7-37.2)	17.5(12.3-24)/17 (12-28)	90/97	16.1/22.1	53.10/62.8	1.6/6.5	NR	6.5/12.6
Pate et al., 2021 [24]	Retrospective cohort	UK	P/S	273/278	NR	NR	NR	NR	31.5/16.9	NR	NR	NR	NR	NR
Stilma et al., 2021 [25]	Retrospective cohort	Netherlands	P/S	438/296	63.8(10.75/65.4(10.25)	72.6/73.3	28.7(4.65)/28.85(7.05)	19.25(8.9)/16.35(9.65)	17/5.7	9.3/7.09	27.1/22.2	NR	NR	3.6/3.7
Al-Hashim et al., 2023 [26]	Retrospective cohort	Jordan	P/S	120/115	55/57*	73.3/67.8	30.3/34.6*	20(18-22)/13(9-20)	NR	3.3/6.1	56.6/60.8	15.8/9.6	14.2/7	10/6.1

Primary Outcomes

**Figure 2:**
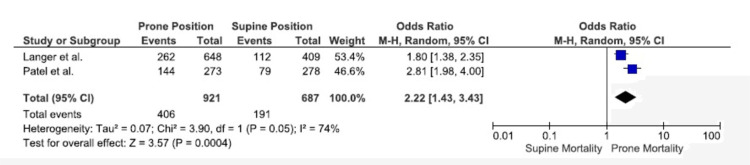
ICU mortality I^2^: heterogeneity, CI: confidence interval, M-H: Mantel-Haenszel method Source: references [21,24]

ICU mortality: The ICU mortality rate was significantly higher in the prone group than in the supine group in combined data from two studies, which included 1608 patients with COVID-19 (OR, 2.22; 95% CI, 1.43-3.43; P = 0.004). The significant heterogeneity with an I^2^ of 74% indicated substantial variability between the two studies (Figure 2) [21,24].

**Figure 3:**
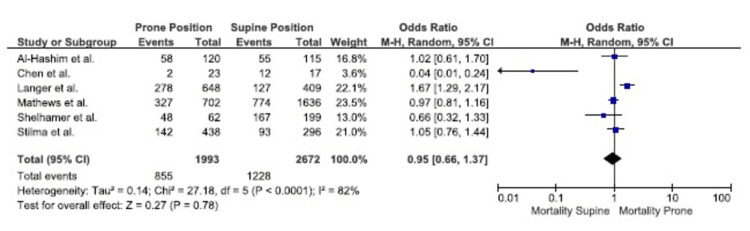
Hospital Mortality I^2^: heterogeneity, CI: confidence interval, M-H: Mantel-Haenszel method Source: references [20-23,25,26]

Hospital mortality: The combined data from six studies that included 4665 patients did not reveal a significant difference in hospital mortality between the prone and supine groups (OR, 0.95; 95% CI, 0.66-1.37; P = 0.78), indicating no significant association between prone positioning and hospital mortality for patients with COVID-19. However, the studies had significant heterogeneity, with an I^2^ of 89% (Figure 3) [20-23,25,26].

**Figure 4:**
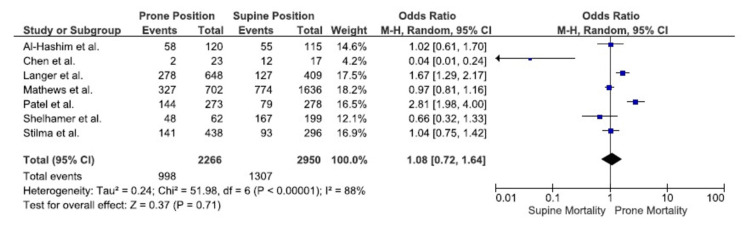
Overall Mortality I^2^: heterogeneity, CI: confidence interval, M-H: Mantel-Haenszel method Source: references [20-24]

Overall mortality: Data combined from all seven studies, including 5216 patients, revealed no difference in overall mortality between the prone and supine groups (OR, 1.08; 95% CI, 0.72-1.64; P = 0.71). The studies had significant heterogeneity (I^2^= 93%) (Figure 4) [20-24].

Secondary Outcomes

**Figure 5:**
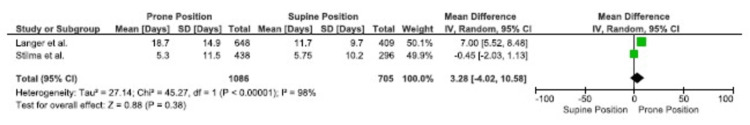
Mechanical Ventilation Days I^2^: heterogeneity, CI: confidence interval, IV: inverse variance Source: references [21,26]

Mechanical ventilation days: Data combined from two studies, including 1791 patients, revealed no difference in mechanical ventilation days between the prone and supine groups (MD, 3.28; 95 %, 4.02-10.58; P = 0.38). The studies had significant heterogeneity (I^2^= 98%) (Figure 5) [21,26]. 

**Figure 6:**
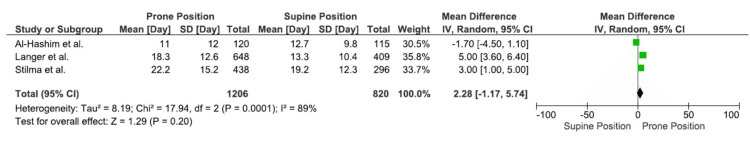
ICU Length of Stay in Days I^2^: heterogeneity, CI: confidence interval, IV: inverse variance Source: references [21,25,26]

ICU length of stay: Data combined from three studies, including 2026 patients, revealed no difference in ICU length of stay between the prone and supine groups (MD, 2.28; 95 %, 1.17-5.74; P = 0.20). The studies had significant heterogeneity (I^2^= 89%) (Figure 6) [21,25,26]. 

**Figure 7:**
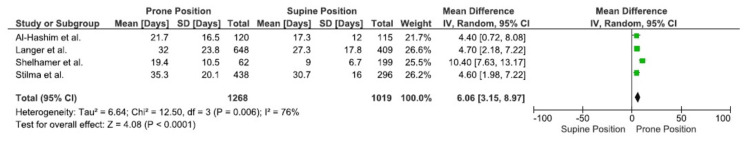
Hospital Length of Stay in Days I^2^: heterogeneity, CI: confidence interval, IV: inverse variance Source: references [21,23,25,26]

Hospital length of stay: Data combined from four studies, including 2287 patients, revealed a prolonged hospital length of stay in the prone group (MD, 6.0; 95 %, 3.15-8.97; P < 0.0001). The studies had significant heterogeneity (I^2^= 76%) (Figure 7) [21,23,25,26].

Discussion

In this meta-analysis, which assessed seven retrospective studies with a total of 5216 patients, no statistically significant difference in hospital mortality or overall mortality was observed between patients with COVID-19 who underwent prone versus supine mechanical ventilation. Based on results from two studies, there was a significantly higher incidence of ICU mortality in the prone group. Based on four studies, the hospital length of stay was significantly longer in the prone group. There was no significant difference between ICU length of stay and days of mechanical ventilation.

There were three studies that independently showed a mortality benefit with prone ventilation for patients with COVID-19. Chen et al.’s analysis concluded that a prolonged prone position is a safe and feasible option to extend survival in patients with COVID-19 [20]. However, the study excluded patients who could not tolerate prolonged prone positioning, which may have introduced bias, particularly if the exclusion had been based on worsening oxygenation or hemodynamic stability. Without more information on the exclusion criteria, determining the potential impact of that choice on the study’s findings is difficult. Additionally, the lack of a clear definition of prolonged prone positioning and intolerance further complicates the interpretation of the results [27]. The study by Shelhamer et al. also had some limitations that could have affected the outcomes [23]. The groups of patients who underwent prone positioning and no prone positioning differed in demographics, comorbid conditions, COVID-19 severity, and treatment provided. These differences could have biased the results toward prone positioning leading to a lower mortality rate. Moreover, the study did not provide details about the duration and frequency of prone positioning and whether the patients who underwent it had any adverse events or complications [28]. The study by Mathews et al. showed a mortality benefit of prone positioning for patients with COVID-19 but also had some caveats. Both groups had the same SOFA scores in the liver/renal categories; however, the prone group had more use of steroids and neuromuscular blockade than the supine group [22]. This is important considering the evidence from a meta-analysis that showed an association between steroid use and lower mortality in patients with COVID-19 pneumonia (0.64 compared to usual care or placebo) [29]. All of these factors could have contributed to the increased mortality seen in the supine group compared to the prone group. 

On the other hand, Langer et al. looked at 1057 patients with COVID-19 who had undergone mechanical ventilation; they saw that prone ventilation was associated with increased mortality (45% vs. 33%). Even though the prone group had more moderate-to-severe COVID-19-associated ARDS than the supine group (90% vs. 77%), both groups had similar SOFA scores [21]. In the study by Stilma et al., there was no significant difference in 28 and 90 days mortality between the prone and supine groups [25]. The study by Hashim et al. showed no significant difference in mortality in all patients with moderate to severe ARDS regardless of prone vs supine position, eliminating the bias of having more severe ARDS in one group over the other [26]. Patel et al. defined prone responsiveness as the maintenance of a mean partial pressure of oxygen (PaO_2_)/fraction of inspired oxygen (FiO_2_) ratio of > 150 over seven days after the first prone episode. In the prone positioning of patients with severe ARDS associated with COVID-19, only 29% had a favorable oxygenation response [24]. In other terms, in a majority of patients (71%) with severe ARDS secondary to COVID-19 pneumonia, the PaO_2_/FiO_2_ ratio did not improve beyond 150 after one week of prone ventilation. 

It is worth mentioning that another study looked at the effect of early prone ventilation in patients with severe ARDS secondary to COVID-19 pneumonia [30]. The authors hypothesized that early prone ventilation would improve outcomes as per findings from the PROSEVA trial. The study included 1714 patients with COVID-19 in the ICU and found no association between early use of prone ventilation and survival in patients with severe hypoxemia on ICU admission. The 30-day mortality rate was not statistically significant in both groups. The mortality was 22.3% for patients who did not receive early-prone ventilation and 26.4% for patients who received early-prone ventilation. This finding contradicts findings from the PROSEVA study and casts doubt on the concept of a mortality benefit from early prone ventilation in patients with COVID-19-associated ARDS.

Our study had some limitations. Retrospective studies are limited by potential biases, such as selection bias and confounding variables, and they cannot definitively determine cause and effect, but only association. Additionally, differences in study design, patient populations, and treatment protocols make comparisons challenging and may have impacted the results. We were not able to stratify the groups based on ARDS severity with the available data.

## Conclusions

While our meta-analysis did not show a mortality benefit to prone ventilation versus supine ventilation for patients with COVID-19, this therapeutic strategy may still have benefits for health factors such as oxygenation and other outcomes. The choice of prone ventilation should be individualized and based on a careful assessment of each patient’s clinical situation, including any physiological evidence that prone ventilation might have specific benefits. We don't recommend performing prone ventilation in all intubated COVID-19 patients.

Furthermore, the limitations of the retrospective studies included in our meta-analysis highlight the need for more controlled studies with standardized protocols to assess the effectiveness of prone ventilation for treating patients with COVID-19. Future studies should also consider stratifying patients based on ARDS severity to better understand the potential benefits of prone ventilation for patients with COVID-19 pneumonia.
